# The Mini-Craniotomy for cSDH Revisited: New Perspectives

**DOI:** 10.3389/fneur.2021.660885

**Published:** 2021-05-06

**Authors:** Jefferson W. Chen, Jordan C. Xu, Dennis Malkasian, Mari A. Perez-Rosendahl, Diem Kieu Tran

**Affiliations:** ^1^Department of Neurological Surgery, University of California, Irvine, Orange, CA, United States; ^2^Neuropathology Division, Department of Pathology, University of California, Irvine, Orange, CA, United States

**Keywords:** chronic subdural hematoma, neurosurgery, dural lymphatics, glymphatics, mini-craniotomy, fenestration, inner membrane

## Abstract

**Background:** Chronic subdural hematomas (cSDH) are increasingly prevalent worldwide with the increased aging population and anticoagulant use. Different surgical, medical, and endovascular treatments have had varying success rates. Primary neurosurgical interventions include burr hole drainage of the cSDH and mini-craniotomies/craniotomies with or without fenestration of the inner membrane. A key assessment of the success or failure of cSDH treatments has been symptomatic recurrence rates which have historically ranged from 5 to 30%. Pre-operative prediction of the inner subdural membrane by CT scan was used to guide our decision to perform mini-craniotomies. Release of the inner membrane facilitates the expansion of the brain and likely improves glymphatic flow.

**Methods:** Consecutive mini-craniotomies (*N* = 34) for cSDH evacuation performed by a single neurosurgeon at a quaternary academic medical center/Level I trauma center from July 2018-September 2020 were retrospectively reviewed. Patient characteristics [age, gender, presenting GCS, GOS, initial CTs noting the inner subdural membrane, midline shift (MLS), cSDH width, inner membrane fenestration, cSDH recurrence, post-operative seizures, infections, length of stay] were extracted from the EMR.

**Results:** Twenty nine patients had mini-craniotomies as primary treatment of the cSDH. Mean age = 68.9 ± 19.7 years (range 22–102), mean pre-operative GCS = 14.5 ± 1.1, mean MLS = 6.75 ± 4.2 mm, and mean maximum thickness of cSDH = 17.7 ± 6.0 mm. Twenty four were unilateral, five bilateral, 34 total craniotomies were performed. Thirty three had inner membrane signs on pre-operative head CTs and an inner subdural membrane was fenestrated in all cases except for the one craniotomy that didn't show these characteristic CT findings. Mean operating time = 79.5 ± 26.0 min. Radiographic and clinical improvement occurred in all patients. Mean improvement in MLS = 3.85 ± 2.69. There were no symptomatic recurrences, re-operations, surgical site infections, or deaths during the 6 months of follow-up. One patient was treated for post-operative seizures with AEDs for 6 months.

**Conclusion:** Pre-operative CT scans demonstrating inner subdural membranes may guide one to target the treatment to allow release of this tension band. Mini-craniotomy with careful fenestration of the inner membrane is very effective for this. Brain re-expansion and re-establishment of normal brain interstitial flow may be important in long term outcomes with cSDH and may be related to the recent interests in brain glymphatics and dural lymphatics.

## Introduction

Chronic subdural hematomas are becoming increasingly common worldwide as the population ages and are more frequently on anticoagulants. It has been estimated that the overall incidence is 1.7 to 20.6 per 100,000 population (1–3). This is a common neurosurgical problem and it has been estimated that it will likely be the most common cranial neurosurgical procedure by the year 2025 (4, 5). Additionally, there are long term sequelae of both operated and unoperated cSDH. In a long-term retrospective study (1990–2015) in Finland, the patients with cSDH had continuous excess mortality up to 20 years after the diagnosis (2). Additionally, the presence of the cSDH has been associated with neuronal degeneration and cerebral atrophy as measured on serial imaging studies. In addition to the local mass effect, this suggests the presence of inflammatory and neurotoxic sequelae of the cSDH with or without surgery (6, 7). There is a high incidence of dementia and other cognitive and emotional sequelae in patients with cSDH (8). Whether this is an expected finding in this age group or whether this is related to the cSDH has been debated (2).

**Figure 1:**
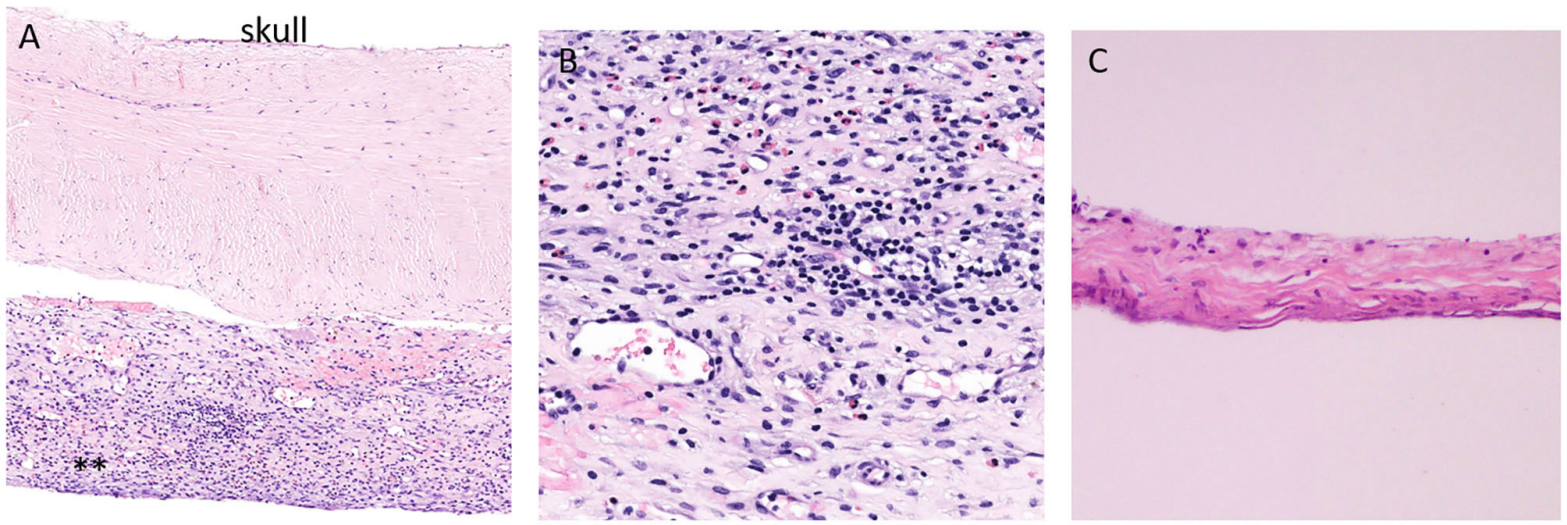
Histopathology of the dura and adjacent outer and inner subdural membrane. **(A)**. H and E stain of the dura with adjacent outer membrane (**). **(B)**. Higher magnification view of the outer subdural membrane demonstrating the high number of inflammatory cells. **(C)**. H and E stain of the inner membrane. Note the thin and avascular and rather acellular appearance.

Blood in the potential space between the arachnoid and the dura elicits a profound inflammatory response generating a highly vascular outer subdural membrane affixed to the dura and an inner collagenous membrane up against the brain pia arachnoid (Figures 1A–C). The outer membrane is the driving force for the growth of the cSDH, and it can be vascular and thick with abundant inflammatory cells (Figure 1B) (9). The inner membrane is thin and relatively avascular and acellular (Figure 1C). Detailed ultrastructural studies have demonstrated the collagen matrix and tight junctions between some of the cells in this membrane (10, 11). The blood break down products are encapsulated between the outer and inner membranes, and as the collection increases in size, the underlying brain parenchyma is compromised and symptoms may ensue depending on the location. This may include headaches, weakness, cognitive decline, and seizures. The outer subdural membrane is very active and has been shown to have high levels of VEGF, inflammatory factors, and autophagy signaling factors (12–14).

Treatment options for cSDH include surgery (15–17), observation and medications such as dexamethasone (12, 18–20) and endovascular obliteration of the meningeal artery (21–25). There have been numerous studies examining the different procedures. In recent years, the burr hole drainage of cSDH has been the most commonly used treatment modality. Advocates argue that the population with the cSDH are generally older patients with many medical co-morbidities and the minimization of the time under anesthesia has many advantages (26–28). However, it is difficult to visualize the septations, vessels, and the inner membrane. There have been several studies demonstrating that endoscopic assisted burr hole drainage of the cSDH aids in the visualization of formed or sequestered hematomas as well as septations and membranes (29).

Critics of craniotomies for cSDH argue that the operative time is higher and there is more morbidity and mortality in comparison to burr hole drainage (15, 30). Reports of craniotomies for cSDH frequently include both large and mini-craniotomies. The reports are mixed as to whether there is fenestration of the inner membrane (5). Manipulation of the inner membrane is done with care to prevent damage to the underlying pia-arachnoid and cortical vessels.

Release of the compressed pia-arachnoid is nicely accomplished *via* craniotomy, evacuation of the chronic subdural hematoma, and fenestration of the inner membrane. We present our experience with this technique in 29 patients (34 craniotomies because of bilateral representation in five) and highlight the nuances of the procedure that may restore and re-establish the brain glymphatic and dural lymphatic circulation. Pre-operative CT scans demonstrating the inner subdural membrane were particularly helpful in guiding the decision to proceed with the mini-craniotomy.

## Materials and Methods

This was a retrospective study of patients who had undergone a mini-craniotomy with evacuation of the cSDH from June 2018 until September 2020 at a single institute. These were consecutive cases performed by the same attending neurosurgeon at a quaternary academic medical center and American College of Surgeons Verified Level I Trauma Center. IRB approval of the study and IRB waiver of consent was obtained because of the retrospective nature of the study. IRB review included ethical review of the study and there were no ethical issues identified. Selection of patients for the mini-craniotomy included the delineation of the inner subdural membrane on the pre-operative CT scan, radiographic findings suggesting break down products of mixed densities, mass effect and neurological symptoms. Additional patients done during this period were excluded from the analysis if they had had a prior cSDH drained at an outside facility or at our facility, thus treatment of persistent or recurrent cSDH were not included in this analysis largely because of the heterogenous nature of the cSDH after prior treatment(s). cSDH drained *via* burr or twist drill holes were not included in this study as the intent of this analysis was to focus on the inner membrane and the effect(s) of opening it. Also, the difficulty and safety associated with opening the inner subdural membrane is examined. Bilateral mini-craniotomies were included in this analysis; however, they were not included if there was burr hole drainage on one side and mini-craniotomy on the contralateral side or if the craniotomies were done at separate settings. Thus, the total number of mini-craniotomies in this evaluation was greater than the number of patients.

### Data Collection

Each case was examined for the patient age, gender, initial GCS, CT and MRI (where available), appearance of cSDH, maximum width of the cSDH, midline shift before and after surgery, mechanism of injury, use of anti-coagulants, fenestration of the inner and outer cSDH membranes, CT resolution of the cSDH, any recurrence of cSDH, improvement if any in the midline shift, any re-operations for recurrence, final GOS, complications (seizures, surgical site infections, new neurological deficits, death within 6 months).

Particular attention was paid to the presence of the identification of the inner membrane on the pre-operative CT scan. This is generally defined as thin hyperdense lines that parallel the skull vault and cerebrum (31). Axial and coronal views were examined for this. Frequently if there is a good deal of mass effect of the cSDH, the limiting inner membrane creates a concave appearance for the cSDH.

### Procedure in Detail

**Figure 2:**
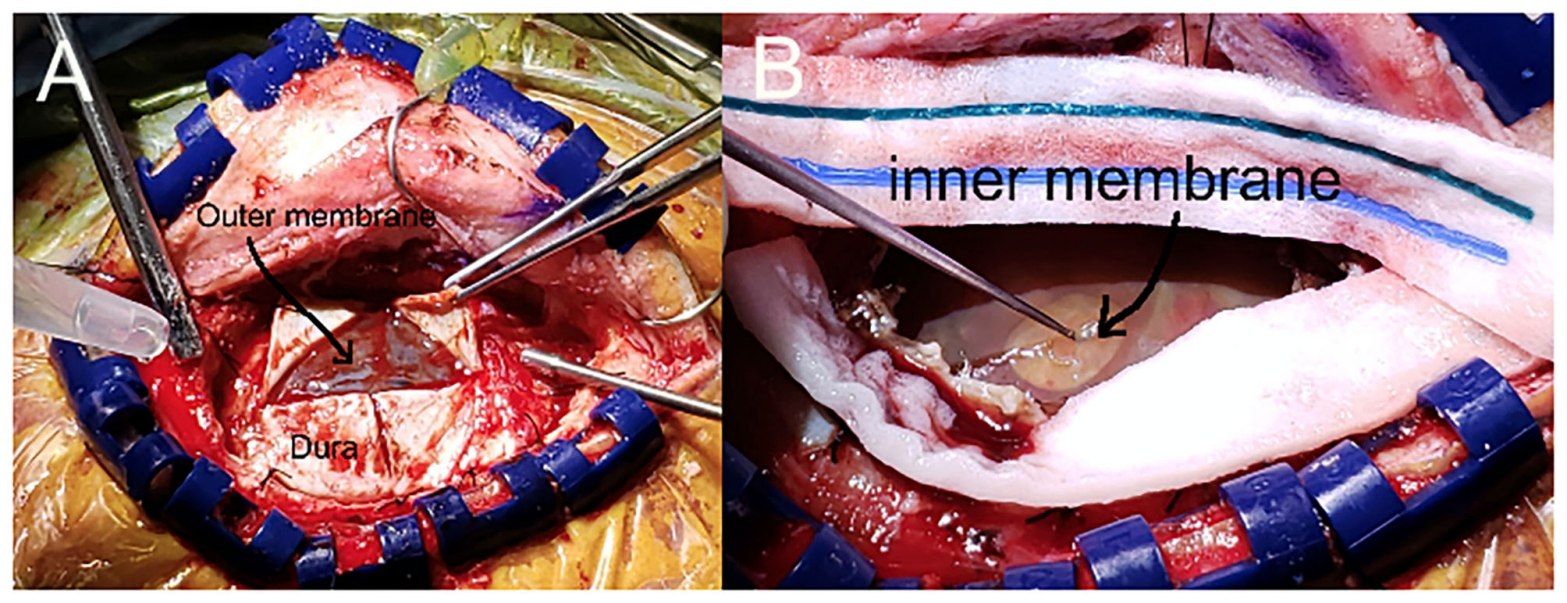
Intraoperative view of the mini-craniotomy demonstrating the inner and outer membrane. **(A)**. Mini-craniotomy demonstrating the retraction of the dural leaflets and the intact outer membrane. **(B)**. The outer membrane has been opened and the cSDH has been removed. Note the gentle lifting up of the opaque yet translucent inner membrane with the microhook that enables the fenestration of the inner membrane.

The mini-craniotomy was planned to be centered over the epicenter of the cSDH at its point of maximum width. The incision was an L-shaped incision and the bone flap, oblong in shape, was 3–4 cm in maximum dimension. Very careful hemostasis was achieved of the epidural space and the dura to ensure that there was no run down of blood at the time of the closure. The dura was opened in a cruciate fashion taking care to note and preserve the outer membrane, which was opened sharply after the dural leaflets were tacked back (Figure 2A). Fenestration of the outer membrane was limited to that which was immediately beneath the dural opening. Edges of the outer membrane were coagulated. No effort was made to “chase” the outer membrane beneath the edges of the bone flap. The liquified cSDH and any subacute and partially formed blood clot was irrigated out with warm sterile normal saline. Any septations or intra-hematoma bridging vessels were coagulated and cut and dissected free. The inner translucent membrane that was adjacent to the underlying pia arachnoid was gently lifted up with the application of low suction and a microhook. The inner membrane was opened sharply with a #11 blade being particularly cautious to avoid any underlying vessels (Figure 2B). The edges of the inner membrane were lifted up and the multiple fenestrations were done in a radial fashion several centimeters away from the entry point using a small Metzenbaum scissors. The edges of the inner membrane were coagulated with bi-polar coagulation to ensure that there was no bleeding into the cavity. The cavity was carefully inspected and irrigated. Particular care was taken to avoid any disruption of the arachnoid membrane so that any of the toxic bi-products of the cSDH did not enter the subarachnoid space. Thus, this helped to mitigate propagation of the inflammatory process of the cSDH. Because the plane was very well-identified between the inner membrane and the arachnoid layer, this was readily accomplished in all cases. The dura was re-approximated in a non-water tight fashion. Gaps in the dura were covered with gel-foam. The bone flap was secured in the usual manner with plating systems. The subdural space was filled with warm normal saline prior to the closure of the skin with a specific effort to eliminate any air in the subdural space. A subgaleal medium hemovac drain was placed and tunneled out through the skin.

After the patient was extubated, a CT scan of the head was done when the patient transitioned from the recovery room to their ICU or floor bed. This occurred within 6 h of reaching the recovery room. The patient was nursed overnight with the head of bed at 0–10 degrees and given a 100% non-rebreather oxygen treatment for 6 h if there was any residual pneumocephalus or if the brain had not fully expanded. Physical and occupational therapy teams worked with the patients starting on post-operative day #1. The hemovac drain was maintained under self-suction until the drain output was <30 cc/24 h or at 3 days (whichever came first) at which point the drain was removed at the bedside taking care to avoid allowing air to track back into the subdural space. An absorbable purse-string suture was placed to close the exit site.

### Additional CT Scans of the Head

Patients that had residual air or blood products had follow up CT scans of the head 24 h later and 1–2 days later until either the residual blood and air were stable or the brain had re-expanded to a near normal state. Patients that did not have any residual subdural fluid/blood or any neurological issues did not have further CT scans of the head. Patients with residual subdural fluid/blood at the time of discharge had f/u CT scans at their 4–6 week routine clinic follow-up.

### Follow-Up

The patients were followed as part of routine care and were given outpatient clinic follow-ups at 1 month, 3 months and 6 months after discharge from the hospital. If the patient did not have full brain re-expansion or if there was any question of residual blood in the subdural space on the last in house CT scan, and if there was a need to restart anti-coagulation, a CT scan was done prior to the resumption of anticoagulants which was 2–3 weeks after the surgery.

## Results

The salient features of our 29 patients that underwent the 34 mini-craniotomies for cSDH are summarized in Tables 1–3. Five patients had bilateral mini-craniotomies. The M:F was 2.6:1. The mean age was 68.9 (S.D. = 19.7) with a range of 22–102 years. Mean initial GCS was 14.5 (S.D. = 1.1) and the mean GOS was 4.9 (S.D. = 0.44). The mean thickness of the cSDH was 17.7 mm (S.D. = 4.2) and the mean mid line shift was 6.75 mm (S.D. = 4.2).

**Table 1:** Patient demographics.

**Patient**	**Age**	**Gender**	**Presenting Symptoms**	**Mechanism of Injury**
1	63	M	Dizziness, imbalance and sleepier	GLF 2 months prior
2	34	M	Confusion	Hx of schizophrenia, hitting himself in the head
3	89	F	Multiple falls, denies symptoms	GLF 1 month prior
4	61	M	Headache, n/v, left sided weakness	No reported trauma
5	22	M	Alcoholic withdraw seizures	Frequent GLF
6*	73	M	Confusion, dizziness	GLF 3 weeks prior
7	78	M	Confusion, incontinence, gait instability	No reported trauma, has hx of NPH s/p VP shunt
8	80	M	Confusion	GLF 2 months prior
9*	44	F	Confusion	Assault 3 weeks prior
10	30	F	Headache	Spontaneous
11	90	F	Confusion	GLF 2 months prior
12	53	M	Progressively worsening headache and unsteady gait	Assault 2–3 months prior
13	39	F	Headache, vomiting	Rollercoaster TBI 1 month prior
14	64	M	Generalized weakness and fatigue	GLF
15	84	M	Fall, denies symptoms	Frequent GLF
16	102	M	Recurrent falls, no symptoms	Frequent GLF
17*	85	M	Remote fall with sdh, enlarged on surveillance imaging, no symptoms	Frequent GLF
18	80	M	Remote fall with surveillance imaging showing increasing size of sdh. No symptoms	GLF 3 months prior
19	77	M	Dizziness and left sided numbness	GLF 1 month prior
20	66	F	AMS and seizures	GLF 2 weeks prior
21	72	M	Confusion	GLF 1 month prior
22	88	F	Slurred speech, right facial droop, right sided weakness	GLF 1 month prior
23*	77	F	Headache	No reported trauma
24*	70	M	Confusion, gait difficulties, word finding difficulties	Fell off bike 2 weeks prior
25	75	M	Broca's aphasia and right sided weakness	Frequent GLF
26	59	M	Left sided headache progressively worsening	No reported trauma
27	79	M	Initially presented due to worsening chest pain, but patient complained of mild headache and had multiple syncopal falls so head CT was done	Frequent GLF
28	91	M	Presented after multiple falls. EMS brought patient in after falling and hitting head on pavement, complained of headache	Frequent GLF
29	75	M	AMS, headache, generalized weakness, trouble with gait	MVC 2 months prior

* *Patients with bilateral cSDH*.

**Table 2:** Characteristics of patients undergoing mini-craniotomies for cSDH.

**Total Patients**	**29**
Mean age (years)	68.9 ± 19.7
Age Range (years)	22–102
M:F ratio	2.46:1
Mean Initial GCS	14.5 ± 1.1
Mean MLS (mm)	6.75 ± 4.2
Mean Max cSDH thickness (mm)	17.7 ± 6.0
Mean ΔMLS immediate postop (mm)*	3.85 ± 2.69
Mean GOS at 6 months follow up**	4.9 ± 0.44

* *Only two patients did not show a change in MLS immediately after surgery out of 29 patients*.

** *Twelve patients had ≥ 1 year follow up either in neurosurgery or neurology clinic. Two patients were lost to follow up because they received their subsequent care in a different healthcare system but were stable at initial follow-up. Three patients were young with complete resolution of their cSDH radiographically and no neurological complaints, thus they requested prn follow-up after the routine 4–6 week clinic visit. The remaining 12 patients were discharged from clinic or given PRN follow-up after 6 months of follow up as their CTs were completely resolved and they had no neurological issues*.

**Table 3:** Surgical factors.

Types of cSDH operated upon	
• cSDH	9
• Subacute cSDH	13
• Acute on cSDH	12
Etiology of cSDH	
• TBI related	22
• Spontaneous	3
• Unknown	4
Fenestration of Inner Membrane	
• Yes	33
• No	1
Types of anticoagulants	
• Eliquis	3
• Coumadin	2
• Anti-plateletes	10

Based on the pre-operative CT scans, nine of the collections were pure cSDH, 13 were considered subacute, and 12 had an acute component in addition to the cSDH (acute on chronic). Fifteen of the patients were on anticoagulants: three on Eliquis, two on coumadin, and 10 on antiplatelet therapy. Twenty-two of the patients had a clear history of TBI in the 1–3 months prior to their presentation to us. Three were spontaneous and 4 were of unknown etiology. Fourteen patients had the obliteration of the subdural space with re-expansion of the brain within 7 days (early resolution of the cSDH). Fifteen had resolution in a delayed fashion (>28 days).

Operative time for the procedure was measured from the time of the skin incision to the final closure. This was 79.5 min (S.D. = 26.11). Where a patient had bilateral craniotomies, the total operative time was divided by two. All of the patients except one had a well-identified inner membrane that was fenestrated at the time of surgery. This was the one patient that did not have a well-defined membrane on the pre-operative CT scan of the head.

The average length of stay after the evacuation of the cSDH was 4.6 days (S.D. = 3.8), which included one patient that remained in the hospital for 22 extra days due to social issues and the need to identify a socially safe place to live. When the analysis was performed without this patient, the average post-operative length of stay was 3.85 days (S.D. = 2.0).

There were no radiographic or symptomatic recurrence of cSDH that occurred during the follow-up period of 6 months. There were no in-hospital deaths. The mean GOS was 4.9 +/−0.44 at the time of discharge from the hospital. One patient was treated for seizures for 6 months (Table 4).

**Table 4:** Measure of surgical outcomes.

Operative time/craniotomy (min)	79.5 ± 26.
Complications (seizures)	3.4% (1)*
Length of stay after surgery (days)	4.6 ± 3.8
Length of stay (after surgery) without outlier**	3.85 ± 2.0

* *One patient experienced a seizure in the immediate post-operative period and was placed on Keppra by neurology for 6 months. The Keppra was then weaned and the patient has been now seizure free for 9 months*.

** *One patient had extended length of stay due to social reasons unrelated to the cSDH surgery or any medical issues. This calculation was done without this patient*.

## Discussion

**Figure 3:**
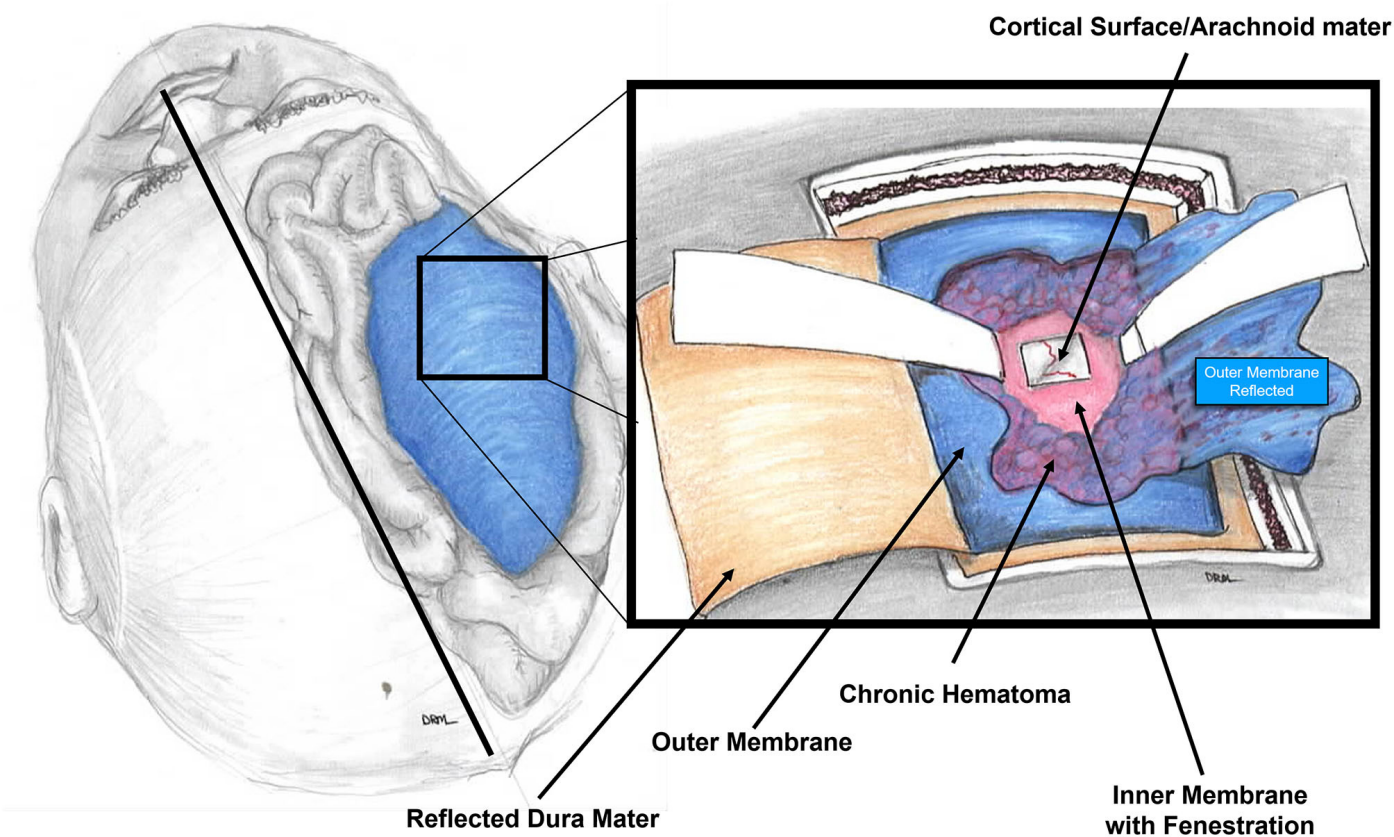
Diagram of the intraoperative view of the mini-craniotomy. The dura is retracted and the outer membrane is depicted with the underlying chronic subdural hematoma. A fenestration of the inner membrane is portrayed. The underlying pia-arachnoid membrane on gross examination is normal appearing.

**Figure 4:**
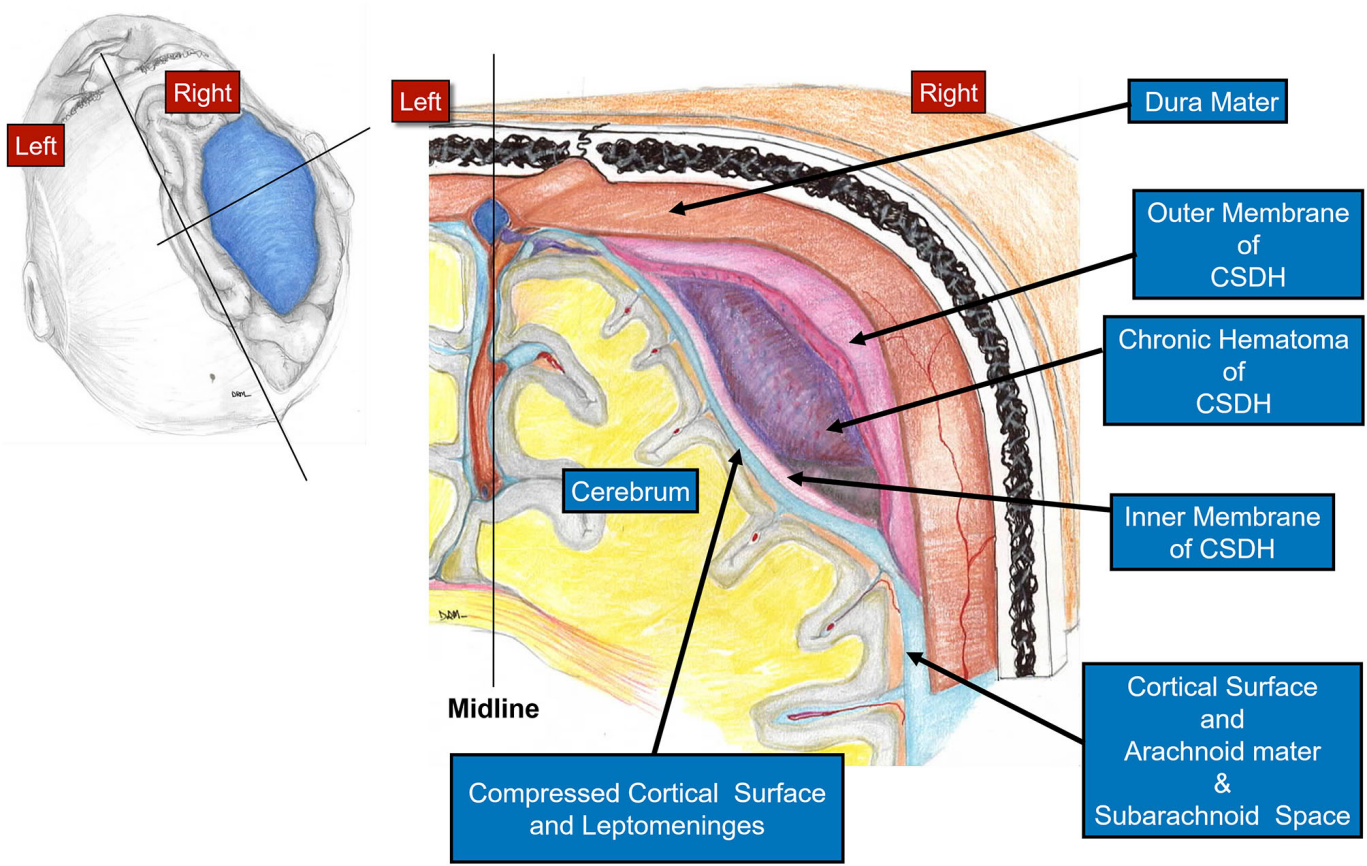
Diagram showing a coronal view through the cSDH. Note the cross-sectional appearance of the layers with the outer membrane adjacent to the dura and the thinner inner membrane pressing upon the pia-arachnoid. The compression upon the underlying cortex affects the flow of interstitial fluid through the brain and the glymphatic system.

In this paper, we present our experience using the mini-craniotomy for the evacuation of cSDH. There have been several reports advocating for this technique primarily because of the improved visualization and the ability to address intracapsular septations and organized clot (16, 17, 32, 33). Figure 3 is a depiction of the relationship between the dura, the outer membrane and the hematoma and the inner membrane and the view that can be afforded *via* a mini-craniotomy. In Figure 4, we can see in the coronal view the encapsulation of the chronic breakdown products of blood as well as the compression upon the underlying cortex. The pia arachnoid is not in direct contact with the toxic blood products as the inner membrane acts as a barrier (12). The specific fenestration of the inner membranes was recently examined and found to result in statistically improved brain expansion when compared to the standard double burr hole technique (34). Recent studies have indicated that one of the most significant factors in the recurrence rate of cSDHs is the amount of pneumocephalus and the rate of expansion of the brain. Early expansion within 7 days was correlated with a decrease in the recurrence rate (35). The immediate mean MLS improvement in our patients was nearly 4 mm.

**Figure 5:**
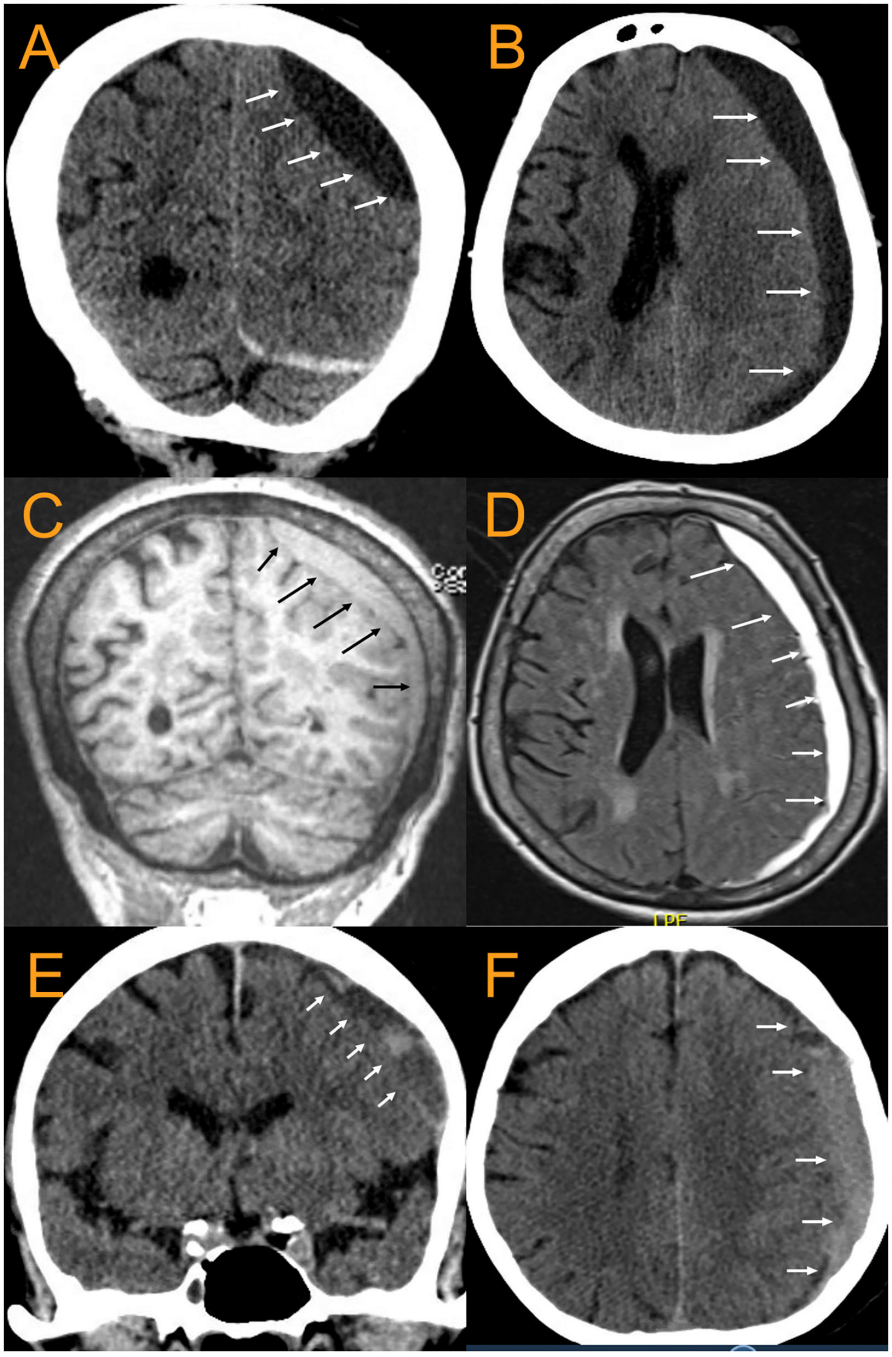
CT and MRI examples of cSDH. 88 yo F with largely chronic SDH with small acute component. **(A)**. coronal CT, **(B)**. Axial CT, **(C)**. MPR coronal MRI, **(D)**. T2-FLAIR Axial MRI. The arrows point to the inner subdural membrane which can be seen delineating a boundary between the cSDH and the pia -arachnoid. **(E,F)**: 79 yoM with subacute cSDH. Once again, the arrows point out the location of the inner subdural membrane.

**Figure 6:**
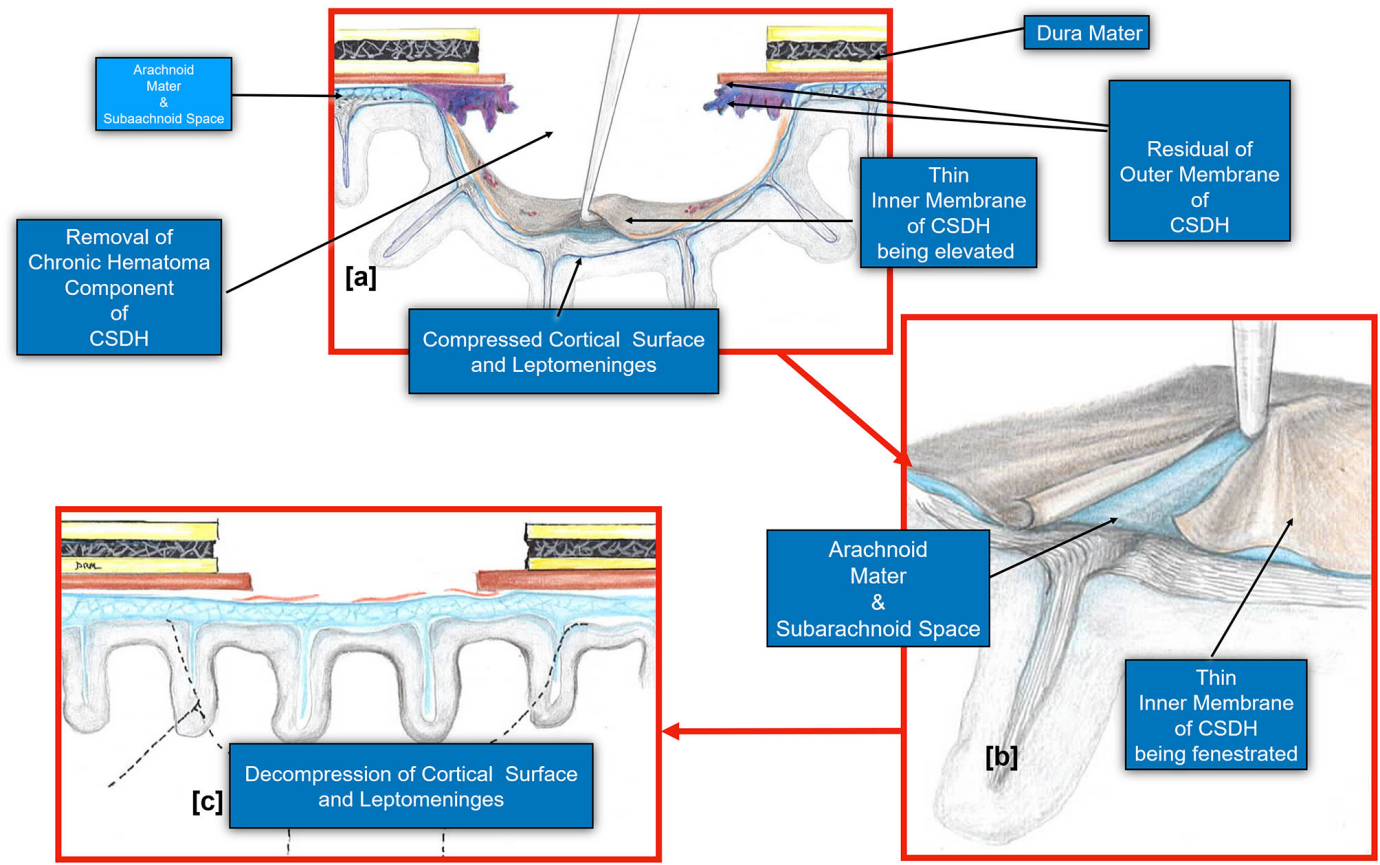
Diagram demonstrating the fenestration of the inner cSDH membrane. **(a)**. The inner subdural membrane is elevated off of the pia-arachnoid using a microhook or by applying suction with a Rhoton #5 sucker. **(b)**. Once this is lifted, a plane is readily developed between the pia-arachnoid and the membrane. A series of cuts may be made extending out radially to complete the fenestration. **(c)**. The fenestration of the inner membrane allows the decompression of the cortical surface and the pia arachnoid. Note that the pia-arachnoid is not opened and rarely is scared to the inner membrane. Occasionally we have noted the accumulation of xanthochromic fluid that has accumulated between the inner membrane and pia arachnoid. This is irrigated out.

**Figure 7:**
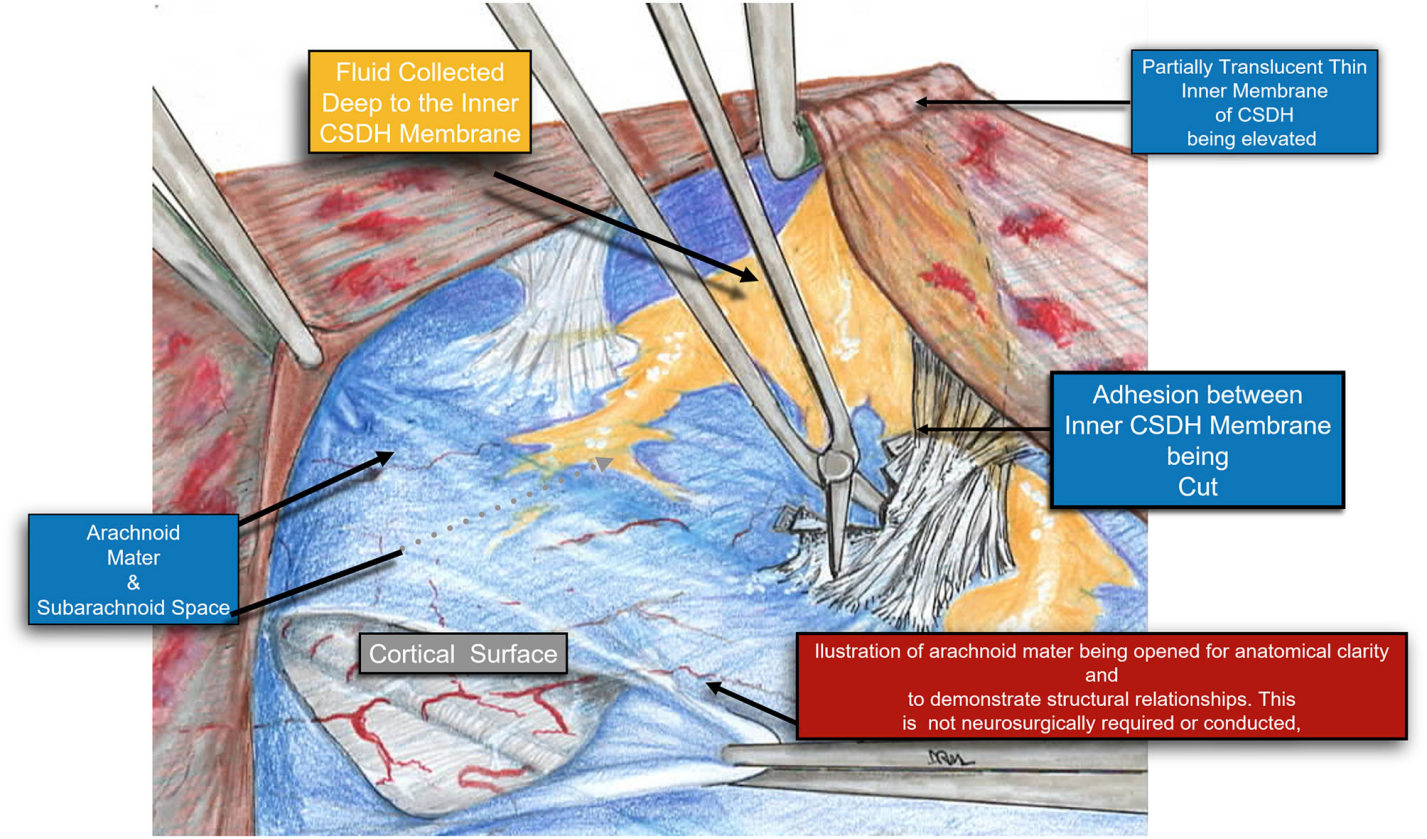
A Higher magnification diagram demonstrating the relationship of the inner cSDH membrane to the pia-arachnoid and the underlying cortex. Adhesions between the pia-arachnoid and the inner subdural membrane were very rare. When encountered these were easily released by cutting them with a microscissors. It is also possible to plan the fenestrations around these areas of adhesion, thus still affecting the release of the tension band of the inner membrane upon the underlying cortex.

Although our study is small and retrospective, we were able to achieve 0% symptomatic recurrence, and 0% mortality. Key to the procedure is the identification of the inner subdural membrane and the wide fenestration of the membrane. This inner membrane can be delineated on pre-operative CT scans and MRI scans (Figure 5). At the time of surgery, we found that the membrane was generally translucent yet xanthochromic in coloration and was pressed up against the pia arachnoid (Figure 2). The thin membrane is seen in Figure 1C which demonstrates its avascular, acellular, and largely collagenous nature. In all of our cases (except the one case where there no inner membrane identified), the inner membrane was not adherent to the cortex and a plane was readily developed between the inner membrane and the pia-arachnoid layer which was not violated. We specifically avoided opening the arachnoid to decrease the chance that the inflammatory breakdown products of the cSDH might aggravate the subarachnoid contents. However, the inner membrane, despite it being thin has tensile strength that is released with fenestration. This allows the brain to re-expand more readily, and this is particularly important in the aged brain which may have underlying atrophy (5, 34). The relationship of the inner membrane to the cortical surface is depicted in Figures 6, 7. No effort was made to remove the inner membrane or strip it off of the pia-arachnoid. Careful fenestration under direct vision (by looking under the bone flap using loupes and a headlight) was done to avoid damage to any vessels. The radial fenestration of the inner membrane allowed for the brain expansion to occur.

**Figure 8:**
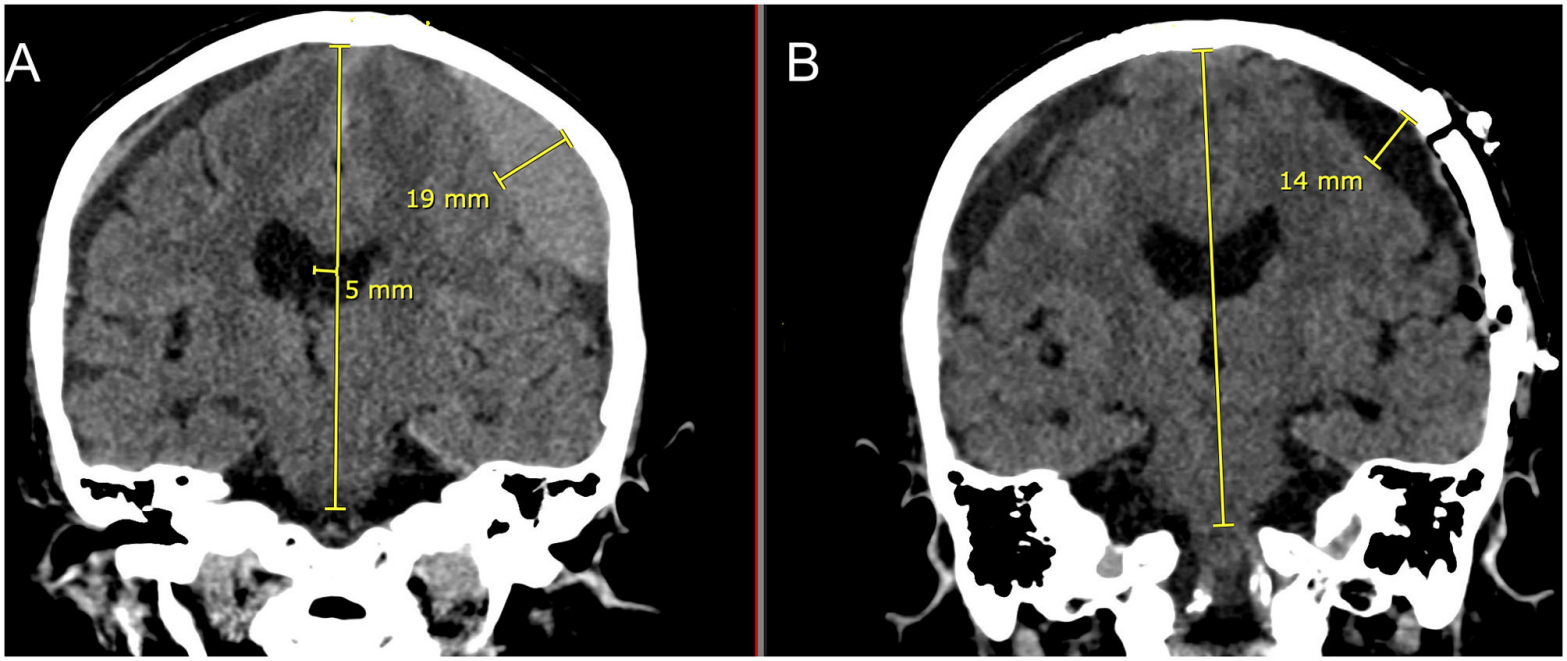
Eighty three yo F who suffered a ground level fall who was on aspirin. She had mild headaches and speech difficulties with a right upper extremity pronator drift. **(A)**: Preoperative coronal CT scan demonstrating a left sided cSDH with midline shift. Note the lentiform shape afforded by the inner subdural membrane that is displacing the underlying cortex. There is a smaller right sided acute on chronic SDH. **(B)**: Post-operative coronal CT scan done within 1–2 h of reaching the recovery room. Note the decrease in the mass effect and midline shift with near complete removal of the cSDH and re-expansion of the brain on the left. There is very little intracranial air. On the right sided there is increased prominence of the subdural space and the acute on chronic subdural hematoma.

Figure 8 is a demonstration of an 83 yo patient with a cSDH that had very nice expansion of the brain after craniotomy and fenestration within 1–2 h of the surgery. In the post-operative CT scan, there is very little intracranial air seen as every effort was made to fill up the entire subdural space with normal saline irrigation during closure. This is particularly important to allow the brain to re-expand in a liquid medium (36).

There is much interest in the long-term sequelae of both operated and non-operated cSDH. Several studies have demonstrated the association of cognitive decline and cerebral atrophy with cSDH (2, 6, 8). Clearly a rather robust inflammatory response of the brain to the initial SDH is elicited and persists (7, 12). Recent trials have been directed at attenuating this response with dexamethasone or atorvastatin (19, 20, 37). There has also been much recent interest in middle meningeal artery (MMA) embolization for the treatment of cSDH. Recent series have demonstrated its efficacy in the stabilization and decrease in size of cSDH after upfront treatment (23–25, 31, 38). The attraction of these embolization techniques is the relative minimal invasive nature which is particularly of concern in the elderly and frail. Most reports are cautious about using MMA up front in cases where there is significant mass effect or neurological deficits. A midline shift of >5 mm or motor strength of 4/5 contralateral to the cSDH has been used as a guideline to avoid MMA embolization (25, 38). As seen in Table 1, all of our patients except for two presented with symptoms/complaints that were concerning enough that they came to our ED. Also, as seen in Table 2, the mean MLS for our patients was 6.75 mm. The MMA embolization has a delayed effect with an average time to resolution of MLS to be 46 days for MLS < 5 mm and 51 days for MLS>5 mm (38). The recent findings with MMA embolization suggest that the mechanism is *via* devascularization of the outer membranes. The finding of embolization material in the inner membrane raises the question of whether or not devitalization of this membrane may also be important in decreasing the size of the cSDH (14, 39).

Clearly the cSDH can exert direct focal pressure on the underlying brain (Figure 4) which may lead to impaired neural dysfunction and long-term neural degeneration. The goal of the surgical treatment whether *via* craniotomy or burr holes is to decompress the brain and to eliminate the toxic effects of the blood break down products. It is difficult to open the inner subdural membrane *via* a burr hole and create a wide fenestration. The benefit of the immediate release of constraints of the inner membrane that are possible *via* the craniotomy are not appreciated with the burr hole method. Over time, because of the thin nature of the inner membrane and because of the outward pressure of the brain, the inner membrane may break up spontaneously. Nonetheless, this is dependent upon the underlying brain. An elderly, somewhat atrophic, brain is unlikely to have the ability to spontaneously disrupt the inner membrane.

The presence of the cSDH likely disrupts the normal flow of brain interstitial fluid because of the local mass effect. There has been much recent interest in the importance of this flow of fluid through the brain *via* the glymphatics and the dural lymphatics (40–42). If the inner membrane is not fenestrated, this itself will act as a constraint on the brain which may continue to affect the glymphatics. Finally, if the brain is unable to expand and if the pia-arachnoid does not contact the dural lymphatics, this may also affect CSF turnover (43, 44). There is increasing evidence that the glymphatic and the dural lymphatic systems are important for clearing potential neurodegenerative toxins such as Tau and Amyloid beta from the brain (43, 45, 46). Aberrancies in their clearance and that of other proteins has been postulated to be a factor in the development of dementia. Further studies into the relationship between cSDH and long term outcomes depending on the type of surgery or procedure performed will help us to better tailor the procedure to optimize the long-term neurological outcomes.

### Limitations of the Study

Limitations of the study include the small sample size and the retrospective nature of the study. This is a single center study examining the results of a single neurosurgeon with follow-up that is limited to 6 months. Additionally, the effect on the long-term cognitive outcome of these patients was not explored. More detailed longitudinal studies of the rate of cerebral atrophy in patients undergoing the mini-craniotomies vs. burr hole drainage need to be done to determine if the theoretical benefits that we propose are realized.

## Conclusion

Mini-craniotomy with careful fenestration of the inner membrane is very effective in the treatment of cSDH with a very low recurrence rate. There are a variety of methods to treat cSDH, the key ones with their advantages and disadvantages are outlined in Table 5. Re-expansion of the brain and re-establishment of normal brain interstitial fluid flow may be important in the success of this procedure and may be related to the recent interests in brain glymphatics and dural lymphatics. This is an area for future research and studies as the number of patients with cSDH and neurodegenerative disorders continues to increase.

**Table 5:** Comparison of the advantages and disadvantages of techniques for treatment of cSDH.

**Method**	**Advantages**	**Disadvantages**
Twist drill hole	May be done at the Bedside under local anesthesia	Difficulty in irrigating out the chronic blood. Does not work well for acute componentsDoes not work well if there are internal septations or well-formed clotsCatheters may easily be misplaced if placed into the subdural spaceAnticoagulants for other medical conditions will need to be held for a finite amount of time
Burr holes	May be done quickly under either General endotracheal anesthesia or local anesthesia with IV sedation The blood products may be washed out *via* irrigation	Difficult to fenestrate inner membrane.Difficult to address acute well-formed componentsDifficult to address internal septations in the cSDHAnticoagulants for other medical conditions will need to be held for a finite amount of time
Mini-Crani	Good visualization of the inner and outer membranes Good access to allow removal of any solid components or to break up any internal septations Good access to allow fenestration of the inner membrane which may enhance expansion of the brain Early expansion of the brain is correlated with decreased recurrence of cSDH	Generally requires GETMore invasive procedure with potentially more discomfort and more risksAnticoagulants for other medical conditions will need to be held for a finite amount of time
Endovascular embolization	Minimally invasive Promising studies have been published recently Multi-center randomized trials comparing to surgery are currently in progress Anticoagulants for other medical conditions do not need to be held	Does not work immediately and may not be applicable if there are significant neurological symptoms/deficits or mass effect

## Data Availability Statement

The datasets generated for this article are not readily available because of patient confidentiality and HIPPA restrictions. De-identified data may be made available upon request. Requests to access the datasets should be directed to Jeff Chen, Jeffewc1@hs.uci.edu.

## Ethics Statement

The studies involving human participants were reviewed and approved by University of California Irvine IRB board. Written informed consent for participation was not required for this study in accordance with the national legislation and the institutional requirements.

## Author Contributions

JC, JX, and DT contributed the writing, conception, and data analysis. DM provided all of the drawn diagrams. MP-R provided the pathology pictures. All authors contributed to the article and approved the submitted version.

## Conflict of Interest

The authors declare that the research was conducted in the absence of any commercial or financial relationships that could be construed as a potential conflict of interest.
